# Doppler Frequency‐Shift Information Processing in WO*_x_*‐Based Memristive Synapse for Auditory Motion Perception

**DOI:** 10.1002/advs.202300030

**Published:** 2023-03-02

**Authors:** Tao Zeng, Zhongqiang Wang, Ya Lin, YanKun Cheng, Xuanyu Shan, Ye Tao, Xiaoning Zhao, Haiyang Xu, Yichun Liu

**Affiliations:** ^1^ Key Laboratory for UV Light‐Emitting Materials and Technology (Northeast Normal University) Ministry of Education 5268 Renmin Street Changchun 130024 P. R. China

**Keywords:** auditory motion perception, azimuth detection, doppler frequency‐shift, memristive synapses, velocity detection

## Abstract

Auditory motion perception is one crucial capability to decode and discriminate the spatiotemporal information for neuromorphic auditory systems. Doppler frequency‐shift feature and interaural time difference (ITD) are two fundamental cues of auditory information processing. In this work, the functions of azimuth detection and velocity detection, as the typical auditory motion perception, are demonstrated in a WO*_x_*‐based memristive synapse. The WO*_x_* memristor presents both the volatile mode (M1) and semi‐nonvolatile mode (M2), which are capable of implementing the high‐pass filtering and processing the spike trains with a relative timing and frequency shift. In particular, the Doppler frequency‐shift information processing for velocity detection is emulated in the WO*_x_* memristor based auditory system for the first time, which relies on a scheme of triplet spike‐timing‐dependent‐plasticity in the memristor. These results provide new opportunities for the mimicry of auditory motion perception and enable the auditory sensory system to be applied in future neuromorphic sensing.

## Graphical abstract

A auditory sensory system with motion perception is demonstrated by using a WO*_x_*‐based memristive synapse. Due to the coexistence of the volatile mode and semi‐nonvolatile mode in the Ar‐plasma‐treated (APT) WO*_x_* memristor, the functions of azimuth detection and velocity detection are realized via implementing the high‐pass filtering and processing the spike trains with frequency shift in both modes, respectively.

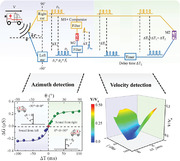

## 1 Introduction

Neuromorphic sensory system, with perception capability similar to its biological counterpart, has attracted significant interest for the human–machine interface, artificial intelligence, and prosthetic applications.^[^ ^1^, ^2^, ^3^, ^4^, ^5^, ^6^, ^7^ ^]^ Sensing and processing are the two fundamental functionalities for the sensory perception, in which advanced sensors with excellent sensitivity have been widely developed in recent years for the sensing function.^[^ ^8^, ^9^, ^10^, ^11^, ^12^ ^]^ However, the sensory information processing in conventional von Neumann architecture usually needs complicated circuits and faces the restriction of high power consumption.^[^ ^13^ ^–^ ^15^ ^]^ In contrast, the human brain is able to seamlessly assimilate and process the sensory stimuli with ultralow power consumption.^[^ ^16^, ^17^, ^18^ ^]^ In particular, the spike patterns generated by all sensory stimuli possess both the spatial and the temporal features.^[^ ^19^, ^20^, ^21^ ^]^ Thus, the ability of processing spatiotemporal information is indispensable for the biological brain to work with high efficiency.^[^ ^22^, ^23^ ^]^ There is an urgent requirement to develop the neuromorphic electronics with capability of processing spatiotemporal information towards more efficient neuromorphic sensory system. Accordingly, two‐/three‐terminal memristors are widely recognized as one of the promising neuromorphic electronics owing to its functional resemblance to the biological synapse.^[^ ^24^, ^25^, ^26^, ^27^, ^28^ ^]^ A variety of synaptic plasticity functions can be emulated in memristors, which provides an ideal platform to biorealisticaly process the spatiotemporal sensory patterns.^[^ ^29^, ^30^, ^31^, ^32^, ^33^, ^34^, ^35^ ^]^

Taking the auditory system as a typical example, it is one of the most efficient sensory systems for human being. The processing of auditory spatiotemporal patterns is crucial to decode and discriminate the auditory information, such as speech recognition, sound localization, and motion perception.^[^ ^36^, ^37^, ^38^ ^]^ Recently, several groups have demonstrated the processing of auditory spatiotemporal patterns in memristive devices and implemented the azimuth detection for static sound localization, which have led to advances in the field.^[^ ^39^, ^40^, ^41^, ^42^ ^]^ However, the auditory system is responsible not only to localize the static sound source, but also to further track the moving sound source, namely, the auditory motion perception.^[^ ^43^, ^44^, ^45^ ^]^ In fact, the sound motion is the only sensory cue available for the perception of mobile objects behind the head. The perception of sound motion is a significant ability for humans to estimate the path of moving sound sources, e.g., avoiding the approaching car.^[^ ^46^, ^47^ ^]^ It is essential to achieve the perception of auditory motion in memristors for neuromorphic auditory system, however, which has rarely been reported to our best knowledge. One challenge is that the detection of sound source velocity is indispensable for auditory motion perception, besides the sound azimuth detection as addressed in previous memristors.^[^ ^39^, ^40^, ^41^, ^42^, ^48^ ^]^ Importantly, the Doppler effect of a mobile sound source is non‐negligible as the most preferred cue for the velocity detection.^[^ ^48^, ^49^, ^50^ ^]^ However, there is still a lack of experimental demonstration of Doppler frequency‐shift pattern processing in memristors.

In this work, we developed an auditory sensory system with motion perception using the WO*_x_*‐based memristive synapses. Therein, taking advantage of the coexistence of volatile mode (M1) and semi‐nonvolatile mode (M2) in Ar‐plasma‐treated (APT) WO*_x_* memristor, the functions of azimuth detection and velocity detection were realized via specific design for circuits. Especially, the Doppler frequency‐shift spatiotemporal information processing was demonstrated using the scheme of triplet‐spike‐timing‐dependent‐plasticity for the first time, which is capable to implement the velocity detection for mobile sound source.

## 2 Results and Discussion

**Figure 1:**
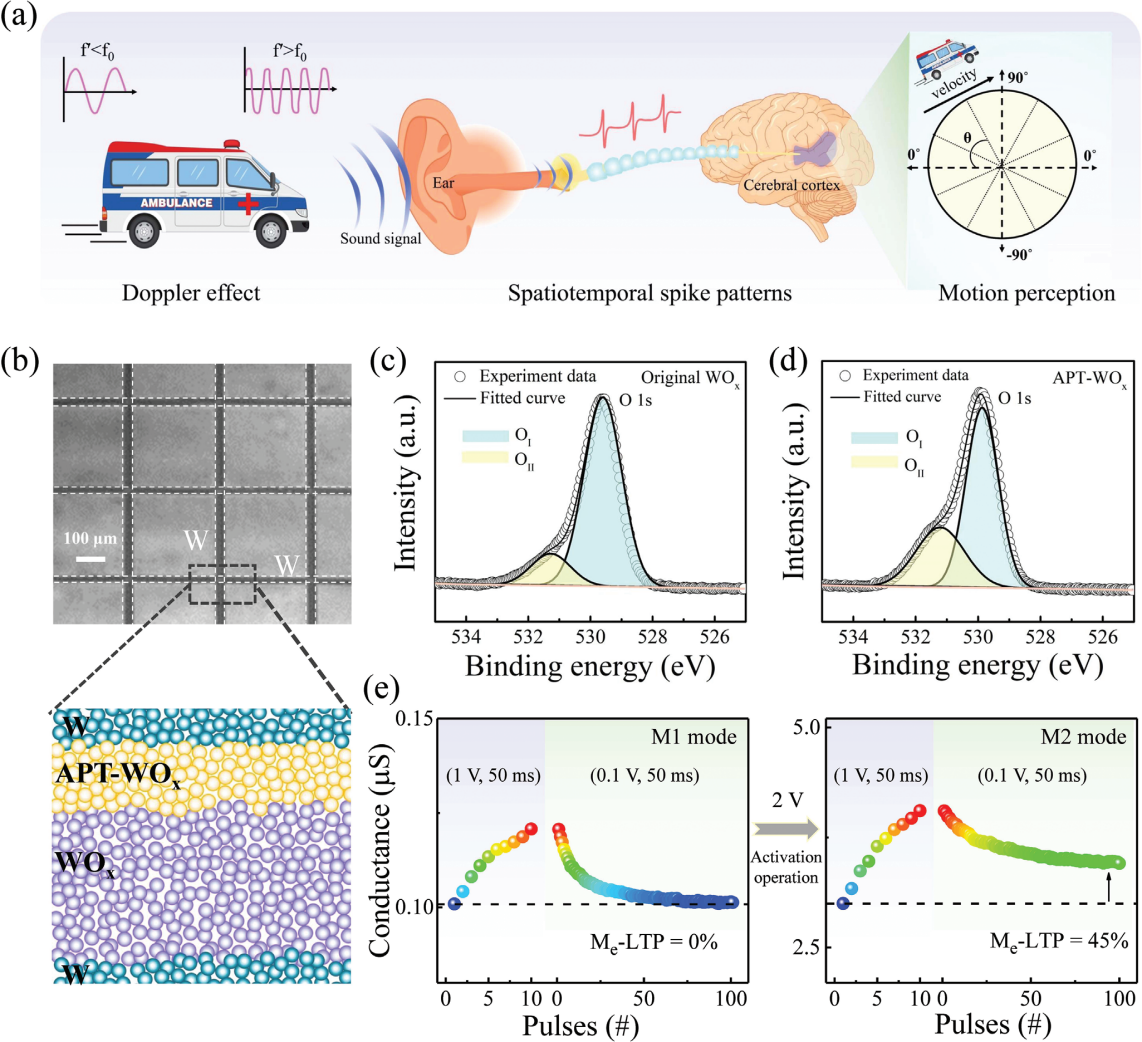
Auditory motion perception emulated in WO*_x_*‐based memristive synapses. a) Schematic of sound motion perception in auditory sensory system. b) The structure of the W/APT‐WO*_x_*/WO*_x_*/W memristor device and the scanning electron microscopy image of the memristor crossbar array. c,d) The XPS of O1s in WO*_x_* film before and after APT. e) Comparison of the conductance decay after the potentiation process in M1 mode (left panel) and M2 mode (right panel). Using an activation operation [+2 V, 50 ms], the transition from volatile M1 mode to semi‐nonvolatile M2 mode occurs under same stimulation [+1 V, 50 ms]. The relaxation process was monitored under a read pulse [+0.1 V, 50 ms].

**Figure** **1**a depicts the motivation for the experimental demonstration of auditory motion perception in memristive hardware. In human auditory system, the sound signals are first received and transformed to spatiotemporal spike patterns in human ears. For a mobile sound source, the frequency of spatiotemporal pattern generally shifts under the Doppler effect owing to the relative motion of sound source and observer.^[^ ^48^, ^51^, ^52^, ^53^ ^]^ The spatiotemporal patterns are then discriminated and processed in the cerebral cortex for motion perception. Therein, the azimuth detection and velocity detection are two critical aspects of spatiotemporal processing in motion perception, which corresponds to the fundamental cues of interaural time difference (ITD) and the Doppler frequency‐shift, respectively.^[^ ^48^ ^]^ Therefore, the processing of ITD and Doppler frequency‐shift information in memristor is the key to realize the auditory motion perception. Figure 1b illustrates the schematic structure of W/WO*_x_*/W memristor employed in current work, which was prepared in crossbar arrays by a sputtering deposition technique (See the Experimental Section). Note that the WO*_x_* layer was treated by Ar‐plasma technique with a power of 10 W for 60 s on its top surface, thereby generating more oxygen vacancies (V_O_) on the top interface of W/WO*_x_*. This is supported by the X‐ray photoelectron spectroscopy (XPS) analysis of Figure 1c,d, which shows the O 1s peak spectra of WO*_x_* film before and after Ar‐plasma treatment (APT). The O 1s peak can be deconvoluted into two components of O_I_ and O_II_ peaks located at 530.1 and 531.5 eV respectively, which are related to contributions of lattice oxygen and the oxygen vacancies. It can be observed that the ratio of O_II_/(O_II_ + O_I_) increased after APT, suggesting more Vo were induced by APT. The XPS results demonstrated a nonstoichiometric, oxygen‐deficient WO_3_ structure and the compositional stoichiometry between “W” and “O” for pristine and APT WO*_x_* are estimated to be about 1:2.6 and 1:2.3, respectively, by the peak area and sensitivity factor of the two elements. Herein, the conductance (*G*) of WO_x_ memristor is regarded as the synaptic weight for synaptic emulation. Interestingly, the WO*_x_* memristor can be switched between two types of memristive modes, the volatile switching mode (M1) and the semi‐nonvolatile switching mode (M2), as illustrated in Figure 1e. More importantly, the functions of azimuth detection and velocity detection can be correspondingly demonstrated under the modes of M1 and M2, which will be discussed in later sections.

The left panel of Figure 1e shows the M1 mode operated in pristine device. The conductance can be gradually enhanced under the stimulation of 10 consecutive positive pulses [+1 V, 50 ms], and then decays to the initial state in the following relaxation process. This suggests the typical behavior of volatile memristive switching in M1 mode, which is usually involved to the implementation of short‐term plasticity.^[^ ^54^, ^55^ ^]^ An activation operation can induce the transition from the mode of M1 to M2 by applying 10 larger positive pulses [+2 V, 50 ms], as shown in Figure 1e. Comparing to the M1 mode, the potentiation conductance in mode M2 would decay to a middle state although experiencing the same stimulation [+1 V, 50 ms], in which the middle state can be retained in long‐term duration.^[^ ^56^ ^]^ It means the transition from short‐term plasticity to long‐term plasticity can be achieved in mode M2, as seen in the right panel of Figure 1e. Such behavior is usually regarded as a clear indicator of second‐order memristor, and similar results are widely addressed in previous works.^[^ ^57^, ^58^, ^59^ ^]^ Herein, both devices in M1 and M2 modes show high reliable and reproducible cycle‐to‐cycle memristive switching except for some acceptable fluctuations, as shown in Figure S1 in the Supporting Information. The different potentiation processes in these two modes also can be seen in the *I–V* curves of memristive switching (See Figure S2 in the Supporting Information). Notably, the large pulse should exceed the threshold voltage of 1.4 V to obtain the transition from M1 to M2 mode, which was shown in Figure S3 in the Supporting Information. Additionally, the transition from mode M2 to M1 mode can be operated by using a deactivation process with 10 negative pulses [−2 V, 50 ms], as shown in Figure S4 in the Supporting Information. Both the conductance change in modes of M1 and M2 can be generally explained by the rearrangement of V_O_s in the WO*_x_* film according to previous memristor models (See Figure S5 in the Supporting Information).^[^ ^60^, ^61^ ^]^

Note that the higher positive pulses [+2 V, 50 ms] in activation process can drive the migration of V_O_s through the whole film, thus forming the conducting channels within the film and obviously increasing the device conductance, as illustrated in Figure S5 in the Supporting Information. Subsequently, the device switches to the mode M2, in which the V_O_s migration prefers to happen near the branches of conducting channel, thus increasing the relative area (*A*) of the conducting channel and device conductance. Specifically, although the lateral diffusion of V_O_s also exists, a part of migrated V_O_s can be retained due to the relatively large switching locations, resulting in the semi‐nonvolatile switching property in mode M2 (as shown in Figure S5 in the Supporting Information). Thereby, the switching mechanism of mode M2 can be understood by the above discussions, while similar model was also reported by Du et al.^[^ ^62^ ^]^ In addition, the deactivation process for switching mode M2 to M1 can be attributed to the rupture of conducting channel under negative pulses.

**Figure 2:**
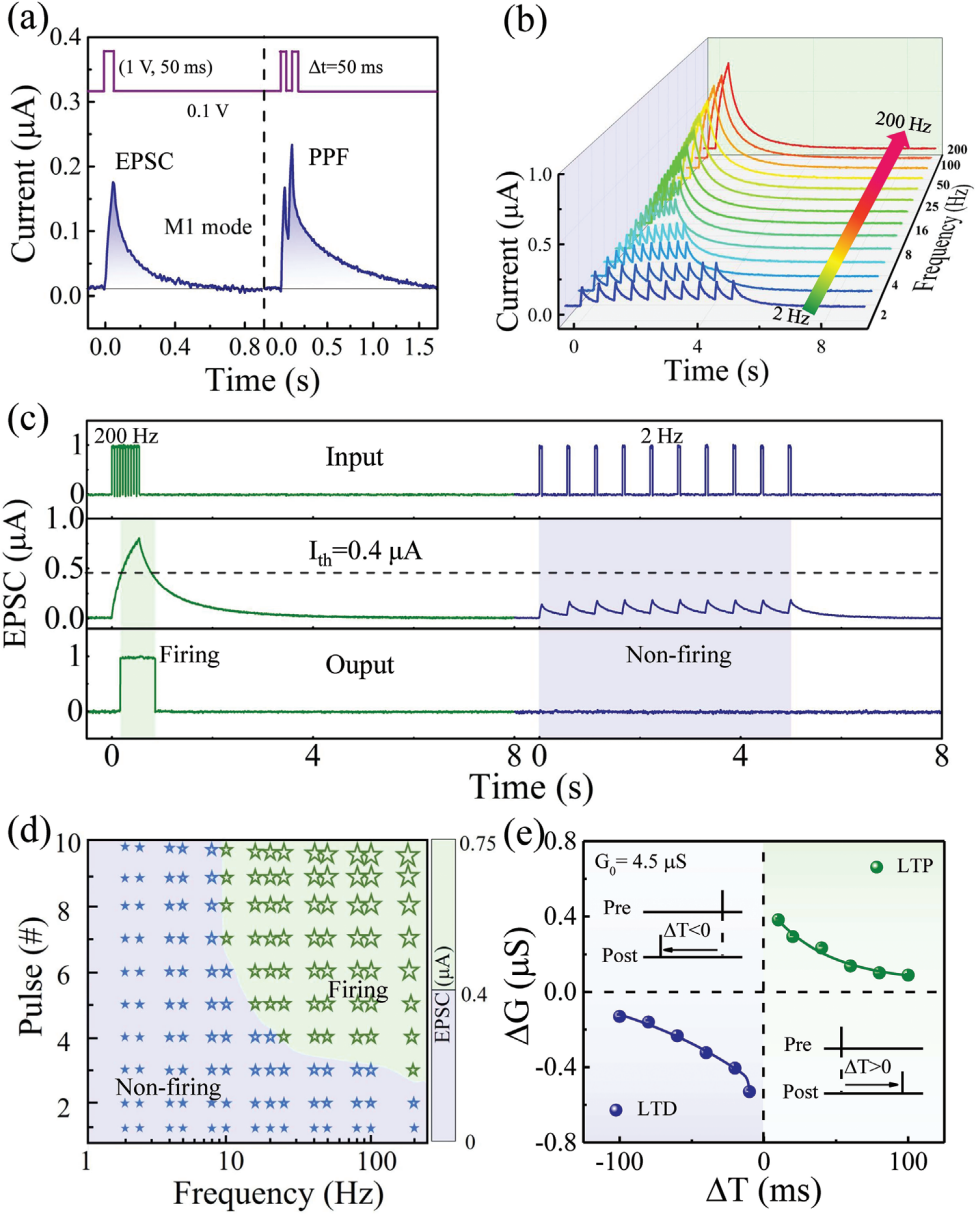
Short‐term and long‐term synaptic characteristics in M1 and M2 modes. a) Synaptic EPSC and PPF functions triggered by a single spike [1 V, 50 ms] and paired spikes in M1 mode. b) Dependence of the EPSC amplitude on the rate of spike trains (2–200 Hz). Ten pulses [1 V, 10 ms] were used to stimulate the device. c) High‐pass filtering and firing under the stimulation with the spike rates of 200 and 2 Hz in M1 mode. d) The summary of experimental conditions for “firing” and “non‐firing” operations by changing the spike number and spike rate. e) The long‐term paired‐STDP emulated in M2 mode.

The short‐term and long‐term synaptic characteristics in the modes of M1 and M2 are first studied in **Figure** **2**, which are the foundation to demonstrate the azimuth detection and velocity detection functions later. Figure 2a shows the current response of memristive device in mode M1 under a single spike [+1 V, 50 ms] and paired spikes. The single spike triggers an abrupt increase in current followed by a decay to initial state within 400 ms. For the paired spikes, the second spike can further enhance the response before the current induced by the first spike decay completely. The above results are similar to the behaviors of the excitatory postsynaptic current (EPSC) and paired‐pulse facilitation (PPF) of the biological synapse, which can be ascribed to the migration and diffusion of V_O_s.^[^ ^57^, ^58^, ^59^, ^62^ ^]^ In particular, the emulation of PPF function means the correlation can be made between the temporal spike pair.^[^ ^63^ ^]^ Furthermore, the PPF function can be extended to the spike‐rate‐dependent plasticity (SRDP) function using the spike trains, as shown in Figure 2b. Each spike train contains 10 consecutive spikes [+1 V, 50 ms] with different spike rate ranging from 2 to 200 Hz. It can be seen that the much larger EPSCs are obtained by increasing the spike rate similar to the SRDP function in biological synapse.^[^ ^64^ ^]^

Through connecting the device to a comparator with certain threshold current *I*_th_, this type of short‐term SRDP is capable to implement the dynamic high‐pass filtering and firing operations for designing the circuit for azimuth detection later. The high‐pass filtering operation is able to avoid the weak signals with the relatively low rate (<10 Hz), while the firing operation can convert the spike train to a single pulse for detecting ITD. Figure 2c shows the examples of high‐pass filtering and firing under the spike rates of 200 and 2 Hz, in which the *I*_th_ of 0.4 µA was selected. The EPSC induced by higher spike rate of 200 Hz is larger than the *I*_th_, thus triggering the “firing” operation and generating an output spike. On the other hand, the EPSC induced by lower spike rate of 2 Hz is too low to trigger the “firing” operation. Figure 2d summarizes the experimental conditions of “firing” operation depending on the spike number and spike rate.

In accordance with the result of Figure 1e, the conductance change (Δ*G*) in EPSC and PPF functions can be transferred from short‐term to long‐term retention in mode M2 (See Figure S6 in the Supporting Information). Importantly, the long‐term spike‐timing‐dependent plasticity (STDP) can be biorealistically implemented by a pair of spikes with temporal correlation, as shown in Figure 2e. The long‐term STDP will be used to process the ITD signals for azimuth detection later. In order to emulate STDP function, the pre‐ and postsynaptic spikes [1 V, 50 ms] are respectively applied on the top and bottom electrode for stimulations. The device conductance of initial state and final state are read before and 1 min after applying the paired pulses, marked as *G*_0_ and *G*_final_ respectively. Herein the Δ*G* is defined as Δ*G* = *G*_final_−*G*_0_. It can be seen that long‐term potentiation (LTP) happens if the presynaptic spike is earlier than the postsynaptic spike (i.e., Δ*t* = *t*_post_ − *t*_pre,_ Δ*t* > 0), while the long‐term depression (LTD) occurs when the postsynaptic spike comes earlier (Δ*t* < 0).^[^ ^65^ ^]^

**Figure 3:**
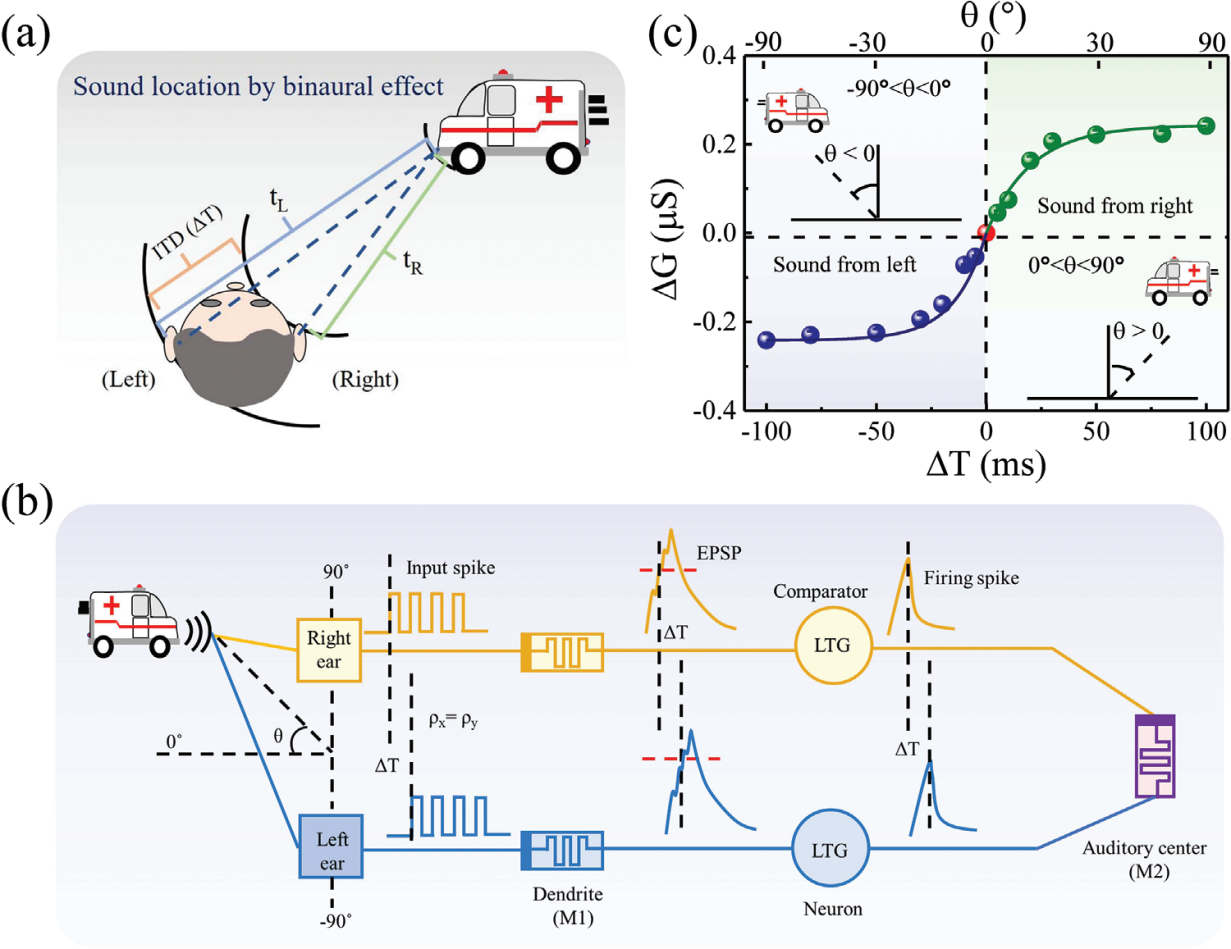
Sound azimuth detection based on WO*_x_* memristors. a) Schematic diagram of sound location by ITD effect in the human ears. b) The circuit design of the ITD‐based azimuth detection using the WO*_x_* memristors. c) Experimental results of Δ*G* as a function of the interaural time difference (ITD) and the sound azimuth *θ*.

Azimuth detection is one of the critical capabilities in auditory system. It is generally achieved by detecting the ITD (Δ*T* = *t*_L_−*t*_R_), where *t*_L_ and *t*_R_ are the times of the sound arriving at the left and right ears, respectively, as descripted in **Figure** **3**a.^[^ ^66^ ^]^ Herein, the combination of high‐pass filtering and firing in mode M1 and paired‐STDP in mode M2 provide the fundamental property to realize the azimuth detection. Figure 3b illustrates the working mechanism of synaptic computation circuit for ITD‐based azimuth detection. The azimuth *θ* is defined as the angle between the line of sight and the line to the sound source location and the time delay Δ*T* can be expressed as Δ*T* = (*k* × *D* × sin*θ*)*/v*, where *D* is distance between the left and right ear, *θ* is the azimuth angle, *v* is the speed of sound, and *k* is the multiplication ratio (See Figure S7 in the Supporting Information). First, the cochlea in both ears receive the sound signals and convert them into two input spike trains with the same rate (*ρ_x_* = *ρ_y_*), while a time delay Δ*T* exists between two ears due to the ITD effect. *ρ_x_* and *ρ_y_* are the spike rate of sound signal in left ear and right ear. The Δ*T* is directly related to the azimuth *θ* as mentioned before. Second, the two spike trains are transmitted to the memristors (dendrite) in mode M1 and two comparators (neuron) in sequence, where the EPSCs are generated following the short‐term SRDP function and converted into a single pulse through the “firing” operation. The weak signals with relatively low spike rate (<10 Hz) are avoided as their EPSC amplitudes are below the *I*_th_ of “firing” operation. Third, two single pulses with Δ*T* are applied on the top and bottom electrode of the memristor in mode M2 (auditory center), thereby producing the long‐term STDP similar to the result of Figure 2e. Figure 3c shows the experimental result of Δ*G* as a function of the ITD Δ*T* and the sound azimuth *θ*. As the Δ*T* changes from 0 to 100 ms, the Δ*G* is positive and increases with Δ*T*, indicating the sound azimuth is 0° < *θ* < 90°; while as the Δ*T* changes from −100 to 0 ms, the Δ*G* is negative and increases with Δ*T*, indicating the sound azimuth is −90° < *θ* < 0°. Figure 3c clearly shows that each Δ*T* corresponds to a specific azimuth *θ*, which means the relative timing‐dependent discrimination is capable for azimuth detection. Previous works with similar design were also reported to realize the azimuth detection for static sound localization.^[^ ^39^, ^40^, ^41^, ^42^ ^]^ The current work will further provide a complete circuit design later for demonstrating both the azimuth detection and velocity detection of mobile sound source.

**Figure 4:**
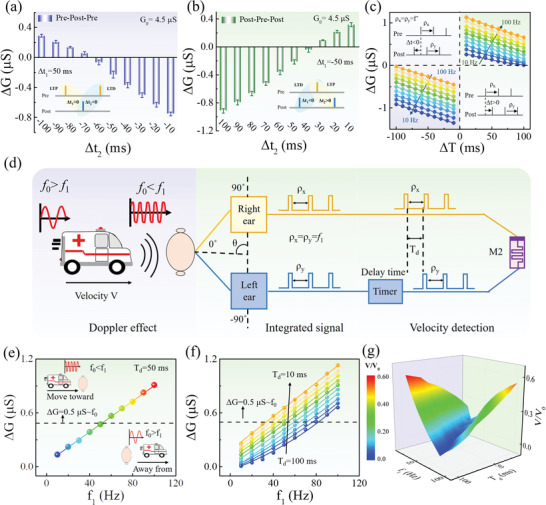
Velocity detection based on WO*_x_* memristors. Triplet‐STDP with asymmetrical spike timing in the a) “pre–post–pre” and b) “post–pre–post” sequences. “pre–post–pre” sequence: Δ*t*_1_ = 50 ms, Δ*t*_2_ is from −10 to −100 ms; “post–pre–post” sequence: Δ*t*_1_ = −50 ms, Δ*t*_2_ is from 10 to 100 ms. c) The dependence of Δ*G* on both the presynaptic spike rate *ρ_x_* and postsynaptic spike rate *ρ_y_*. The schematic of the operation signal is shown in the inset, in which ten pairs of presynaptic spike and postsynaptic spikes were used. d) The phenomenon of Doppler effect and the circuit design of Doppler velocimeter using the memristor in M2 mode. e–g) Doppler frequency‐shift information processing by using the Doppler velocimeter. (e) Typical result of Δ*G* as a function of the spike frequency *f*_1_ in this case of *T*_d_ = 50 ms, and the reference frequency *f*_0_ is selected by using Δ*G* = 0.5 µs. f) The dependence of *f*_1_ and Δ*G* with different delay times *T*_d_ from 10 to 100 ms. g) Experimental results of *V*/*V*_0_ under the conditions with different *f*_1_ and *T*_d_ based on the Doppler velocimeter by using the Equation (1).

The demonstration of velocity detection can be realized by emulating the Doppler effect using our memristor in mode M2. The long‐term SRDP, as the critical function for emulating the Doppler effect for velocity detection, can be correspondingly operated in mode M2. The basic behavior that higher (lower) spike rate results in larger (smaller) long‐term Δ*G* can be seen in Figure S8 in the Supporting Information. Herein, in order to better implement the Doppler frequency‐shift information processing, the long‐term triplet‐STDP as another type of typical SRDP is specifically demonstrated in this work, as illustrated in **Figure** **4**a–c. Comparing to the standard paired‐STDP, triplet‐STDP contains a third spike either presynaptic or postsynaptic.^[^ ^59^, ^67^, ^68^, ^69^ ^]^ The relationship between the paired term and triplet term contributions provides the multiplicative correlations between presynaptic and postsynaptic activities. Therefore, triplet‐STDP possesses advantages over pair‐STDP to demonstrate spiking rate‐based synaptic plasticity, such as rate‐based Bienenstock–Cooper–Munro (BCM) learning rule and high‐order spatiotemporal processing function. For the “pre–post–pre” triplet (Figure 4a), the LTP process is induced by the first‐spike pairing (“pre–post”, Δ*t*_1_ > 0), which is followed by an LTD process induced by the second spike pairing (“post–pre”, Δ*t*_2_ < 0). For the “post–pre–post” triplet (Figure 4b), the LTD process is activated before the LTP process. Each spike applied on the memristor consists of a single pulse (1 V, 50 ms). In the “pre–post–pre” case, Δ*G* transformed from potentiation to depression as the interval of the LTD process (Δ*t*_2_) increased from −100 to −10 ms whereas the interval of the LTP process (Δ*t*_1_ = 50 ms) was kept constant. Similarly, in the “post–pre–post” case, the potentiation of Δ*G* enhanced with decreasing Δ*t*_2_ and while keeping Δ*t*_1_ = −50 ms constant. Therefore, the spike‐timing intervals (Δ*t*_1_ and Δ*t*_2_) play a critical role in the competition of LTP and LTD, determining the potentiation or depression of synaptic weight (*G*).

Figure 4c clearly shows the dependence of LTP/LTD in STDP on the pre‐/postsynaptic spike rate ((*ρ_x_*/*ρ*_y_) and the timing interval Δ*T* between pre‐ and postsynaptic spike. Herein, ten pairs of pre‐/postsynaptic spikes were used in the measurement, whereas the pre‐/postsynaptic frequency is defined by *ρ_x_* = 1/Δ*t*_pre–pre_ and *ρ_y_* = 1/*Δt*_post–post_, as schematically described in the inset of Figure 4c. It can be seen that both the LTP (Δ*T* > 0) and LTD (Δ*T* < 0) values increase as the spike frequency increasing from 10 to 100 Hz for each fixed |Δ*T*|, and they increase with decreasing the |Δ*T*| for each fixed spike frequency. This means the Δ*G* of synaptic weight can be modulated by both the spike frequency and Δ*T*, which is an essential feature of triplet‐STDP in biological synapse.^[^ ^67^ ^]^ Importantly, the triplet‐STDP provides a good platform to process the Doppler frequency‐shift information and realize the velocity detection in relative motion. Progress have been made to mimic triplet‐STDP by memristors in literatures and our previous work,^[^ ^59^, ^68^ ^]^ however, the demonstration of Doppler effect relying on triplet‐STDP has not been addressed yet.

Figure 4d schematically illustrates the phenomenon of Doppler effect and the design principle of a Doppler velocimeter using our memristors. Doppler effect is generally observed in the relative motion between a mobile source and an observer, in which the wave frequency increases/decreases when the source moving forward/backward.^[^ ^48^, ^51^, ^52^, ^53^ ^]^ Doppler velocimeter is usually operated by measuring the value of frequency‐shift in Doppler effect. As addressed in literatures,^[^ ^70^ ^]^ the Doppler velocity can be calculated by considering the relationship between the Doppler velocimeter (observer) and the moving object as follows

(1) $$\left\{\begin{array}{c} V=\frac{f_{1}-f_{0}}{f_{1}+f_{0}}V_{0}\;\;\;\mathrm{move}\;\mathrm{toword}\\ V=\frac{f_{0}-f_{1}}{f_{1}+f_{0}}V_{0}\;\;\;\mathrm{away}\;\mathrm{from} \end{array}\right.$$

where *f*_0_ is the reference wave frequency emitted from Doppler velocimeter, *f*_1_ is the received wave frequency of Doppler velocimeter, *V*_0_ is the sound velocity in the medium, *V* is the velocity of moving source. The detailed measurement diagram and description of the calculation equation is shown in Figure S9 in the Supporting Information. According to the Equation (1), since both the *f*_0_ and *V*_0_ are the constant values, the source velocity can be detected by only calculating the received frequency *f*_1_ in the Doppler velocimeter. The Equation (1) considers the particular situation that sound source is moving straight ahead to the Doppler velocimeter, that is, the azimuth *θ* is 0°.

A simple Doppler velocimeter is designed based on the principle of Equation (1) as schematically illustrated in Figure 4d, which includes one memristor in mode M2 and some peripheral circuits. Herein, the particular situation is first considered that the azimuth *θ* is 0°, in which the measured velocity *V* is only related to the spike frequency *f*_1_ generated by the relative motion. First, with the same first step as that of Figure 3b for azimuth detection, both ears also receive the sound signals and convert them into two input spike trains with the same rate (*ρ_x_* = *ρ_y_*) in Figure 4d. The spike rates of *ρ_x_* and *ρ_y_* in left and right ears corresponds the frequency *f*_1_ in Equation (1). Second, before the spike trains transmitting to the memristor, a fixed time difference *T*_d_ is set in the spike train of right ear using a timer. This makes sure the memristor in mode M2 could be implemented following the triplet‐STDP function. Third, two spatiotemporal spike trains with the frequency *f*_1_ and a fixed delay time *T*_d_ a are applied on the top and bottom electrode of the memristor in mode M2 for auditory information processing. Eventually, the velocity of sound source can be achieved by calculating the frequency *f*_1_ through the correlation of spike rate and Δ*G* in triplet‐STDP result.

Figure 4e shows an example of triplet‐STDP result in order to validate the feasibility of the above working mechanism for velocity detection. In this case the delay time *T*_d_ is fixed as 50 ms. It can be seen that the Δ*G* monotonically increases as the spike frequency *f*_1_ increasing from 10 to 100 Hz, which means the *f*_1_ can be confirmed by detecting the Δ*G* value. Furthermore, the reference frequency *f*_0_ is selected by using Δ*G* = 0.5 µs as the threshold value, while the conditions of *f*_1_ > *f*_0_ and *f*_1_ < *f*_0_ represent that the sound sources move forwards/backward to the Doppler velocimeter, respectively. Therefore, the ratio of sound source velocity to sound velocity (*V*/*V*_0_) can be evaluated through the Equation (1) by confirming the *f*_1_ value, as illustrated in Figure 4e. Additionally, Figure 4f shows dependence of *f*_1_ and Δ*G* with different delay times *T*_d_ from 10 to 100 ms. The modulation of *T*_d_ is beneficial to extend the measurement range of Δ*G*. For instance, the Δ*G* ranges from 0 to 0.65 µs in the case of *T*_d_ = 100 ms, while it ranges from 0.25 to 1.15 µs in the case of *T*_d_ = 10 ms. Figure 4g summarizes the results of *V*/*V*_0_ under the conditions with different *f*_1_ and *T*_d_ using this Doppler velocimeter, which verifies the capability of source velocity detection in our memristor via processing Doppler frequency‐shift information.

**Figure 5:**
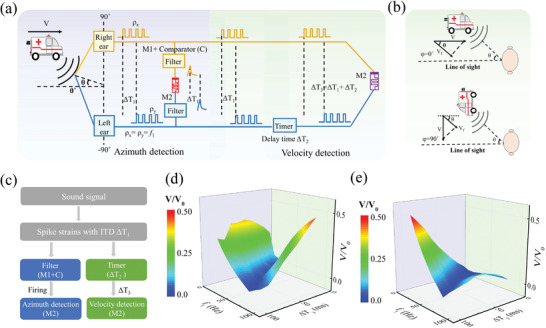
Auditory motion perception based on WO*_x_* memristors. a) Schematic diagram of the complete memristive circuit for auditory motion perception including the azimuth detection (light bule background) and the velocity detection (light yellow background) detections for the auditory motion perception. b) Two general conditions that source velocity is parallel (*φ* = 0°) or perpendicular (*φ* = 90°) to the line of sight. c) Flowchart of information processing using the memristor‐based auditory sensory system. Experimental results of velocity detection with source velocity d) parallel (*φ* = 0°) and e) perpendicular (*φ* = 90°) to line of sight by using the Equation 2.

**Figure** **5**a illustrates the complete memristive circuit for auditory motion perception by combining the designs in Figures 3b and 4d. The system includes the azimuth detection part (light bule background) and velocity detection part (light yellow background), which enables the implementation of these two functions simultaneously. The image of the setup demonstrating the azimuth detection and velocity detection was shown in Figure S10 in the Supporting Information. A general case is considered that the sound source moves towards the observer with a certain azimuth *θ*, in which the direction of source velocity is parallel (*φ* = 0°) or perpendicular (*φ* = 90°) to the line of sight, as illustrated in Figure 5a,b, respectively. For these two conditions, the perceived velocity *V*_f_ of Doppler velocimeter (observer) can be described as

(2) $$\left\{\begin{array}{c} V_{f}=\left[\frac{f_{1}-f_{0}}{f_{1}+f_{0}}V_{0}\right]*\cos\left(\theta +\varphi \right)\;\mathrm{move}\;\mathrm{toword}\\ V_{f}=\left[\frac{f_{0}-f_{1}}{f_{1}+f_{0}}V_{0}\right]*\cos\left(\theta +\varphi \right)\;\mathrm{away}\;\mathrm{form} \end{array}\right.$$

where *V*_f_ is the fractional velocity of the sound source along the direction to the observer, the sum of *θ* and *φ* means the angle between the direction of velocity and the line to the observer.

Figure 5c shows the flowchart of information processing using the memristor‐based auditory motion perception. The processing procedures can be described as follows: (i) the ears receive the sound signals and convert them into two spike strains with the same frequency *f*_1_ (from 10 to 100 Hz) and a certain ITD Δ*T*_1_ (from 0 to 100 ms); (ii) In azimuth detection part, the spike strains are further converted to two single pulses using a dynamic filter (a memristor in mode M1 and a comparator), which generates the paired‐STDP result to estimate the azimuth *θ* (from −90° to 90°); the experimental result of azimuth detection can be referred to Figure 3c; (iii) In velocity detection part, the spike train of left ear is delayed for Δ*T*_2_ using a timer, thus producing the total time difference between these two signals as Δ*T*_3_ = Δ*T*_1_ + Δ*T*_2_. According to the Equation (2), the value of *V*_f_/*V*_0_ can be first obtained following the triplet‐STDP result, and then the *V*/*V*_0_ are calculated by considering *V*_f_ = *V*cos(*θ*+*φ*). Figure 5d,e illustrates the experimental results of velocity detection in the two conditions that source velocity is parallel (*φ* = 0°) or perpendicular (*φ* = 90°) to the line of sight, verifying the capability of our memristor system for auditory motion perception. Our works implement both the azimuth detection and velocity detection functions, which is absent in the previous works (see Table S1 in the Supporting Information for comparison), suggesting an effective strategy to facilitate auditory sensory system for practical application.

## 3 Conclusion

In conclusion, we demonstrated the auditory sensory system with the functions of azimuth detection and velocity detection based on WO*_x_*‐based memristive synapses. The WO*_x_*‐based memristor can present two modes of memristive switching, i.e., M1 (volatile switching) and M2 (semi‐nonvolatile switching), which can be attributed to the rearrangements of *V*_O_ *s* in the Ar‐plasma‐treated (APT) WO*_x_* film. Importantly, the azimuth detection and velocity detection of mobile sound source were emulated by utilizing the dynamic features of M1 and M2 modes, including the high‐pass filtering and firing in M1 mode and the Doppler frequency‐shift information processing relying on triplet‐STDP in M2 mode. Furthermore, our experiments demonstrate that the proposed auditory sensory system, combined with azimuth and velocity measurements, is capable of paralleling the auditory motion perception of our human brain. It is believed that our study makes a progress towards artificial auditory sensory system for novel architectures of hardware artificial intelligence and paves the way for the application of memristors to neuromorphic sensing

## 4 Experimental Section

### Device Preparation

Memristors with a W/WO*_x_*/W sandwich structures were fabricated on SiO_2_/Si substrates and patterned into a crossbar array with a junction area of 50 × 50 µm^2^ using a metal mask, as shown in Figure 1b. On the SiO_2_/Si substrate, W bottom electrodes were deposited using W target by radio frequency sputtering of 30 W for 5 min, and the WO*_x_* layer was prepared by radio frequency sputtering of 100 W using a metal WO_3−_ *_x_* target at 200 °C for 20 min. The WO*_x_* film was prepared using argon:oxygen = 1:3 with a pressure of 2 Pa. Prior to the deposition of W top electrodes, ultrapure Ar was used as the sputtering gases to treat WO*_x_* surface with sputtering power of 10 W for 60 s. Finally, W top electrodes were deposited on the top of the film to act as electrodes by sputtering.

### Electrical Measurements

Memristive properties were measured using a self‐built test system comprising a sourcemeter (2636A, Keithley), arbitrary function generator (3390, Keithley), oscilloscope (TDS 2012B, Tektronix), and probe station (TTPX, Lake Shore). A bias voltage was applied on the W top electrode and W bottom electrode was grounded. The positive direction of the bias voltage was defined such that the current flowed from the top to the bottom electrode. To measure the EPSC, the memristor was connected with a load resistor *R*_load_ of 1 MΩ in series, and the voltage drop across the *R*_load_ was monitored by an oscilloscope. Then, the monitored voltage was converted to the current flowing through the memristor. To implement pair‐ or triplet‐STDP, each pre‐ or postsynaptic spike applied to the top or bottom electrode was composed of a single spike with amplitude *V*+/*V*− = 1 V/−1 V and a width of 50 ms. The initial and final conductance states of the device (*G*_0_ and *G*_final_) were readout using a small pulse (0.1 V, 50 ms) before and after applying the programmable pulses, and the conductance change was defined as Δ*G* = *G*_final_ − *G*_0_. Both the writing and reading of the memristor were performed in pulse mode.

## Conflict of Interest

The authors declare no conflict of interest.

## Supporting information

Supporting Information

## Data Availability

The data that support the findings of this study are available from the corresponding author upon reasonable request.
