# Supplementary material for: Doppler Frequency‐Shift Information Processing in WO x ‐Based Memristive Synapse for Auditory Motion Perception
Source: Adv Sci (Weinh). 2023 Mar 2;10(13):2300030. doi: 10.1002/advs.202300030 (PMC10161103; doi:10.1002/advs.202300030)
Supplement: Supplementary file 1 — Supporting Information [file ADVS-10-2300030-s001.pdf]

ADVANCED SCIENCE
Open Access

# Supporting Information

for *Adv. Sci.*, DOI 10.1002/advs.202300030

Doppler Frequency-Shift Information Processing in $WO_x$-Based Memristive Synapse for Auditory Motion Perception

*Tao Zeng, Zhongqiang Wang*, Ya Lin*, YanKun Cheng, Xuanyu Shan, Ye Tao, Xiaoning Zhao, Haiyang Xu* and Yichun Liu*

# Supporting Information

**Title: Doppler frequency-shift information processing in $WO_x$-based memristive synapse for auditory motion perception**

*Tao Zeng, Zhongqiang Wang*, Ya Lin*, YanKun Cheng, Xuanyu Shan, Ye Tao, Xiaoning Zhao, Haiyang Xu* and Yichun Liu*

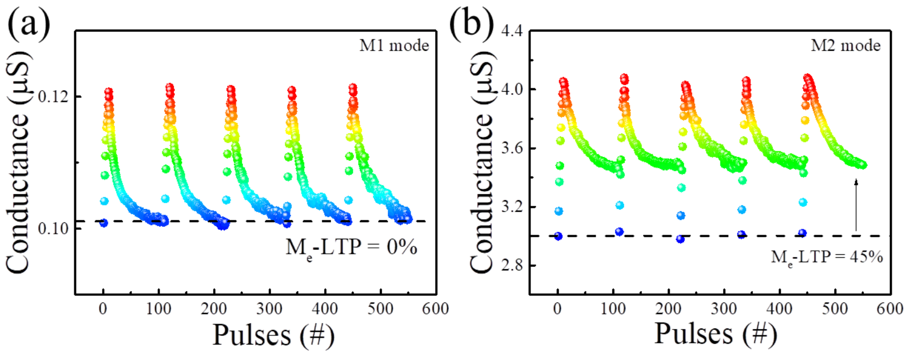


Figure S1. The reproducible properties of memristive synapse in (a) M1 and (b) M2 mode, respectively. The potentiation and relaxation processes were obtained under the stimulation [+1 V, 50 ms] and read pulse [+0.1 V, 50 ms], respectively.

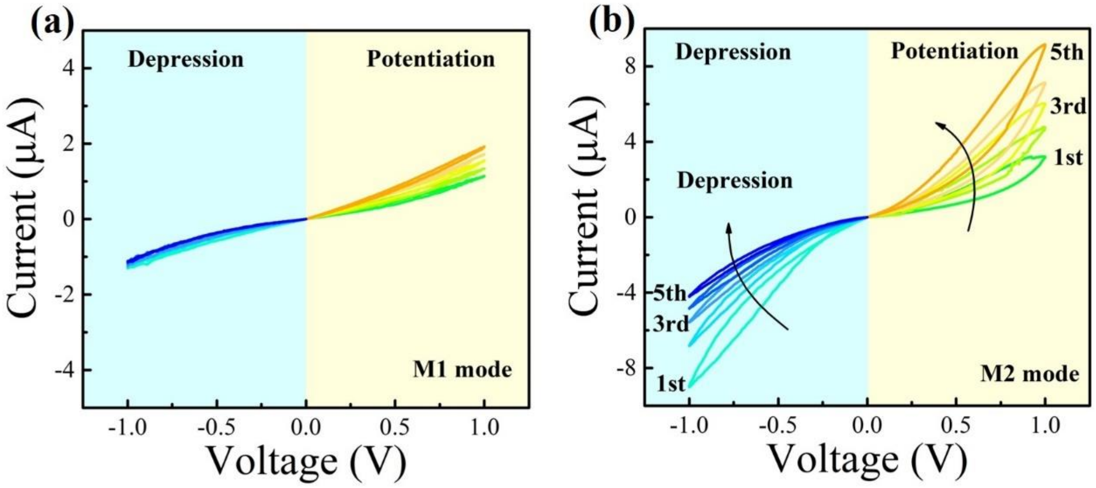


Figure S2. I-V curves of memristive switching in (a) M1 mode and (b) M2 mode. The current can continuously increase/decrease with the positive [0 V → 1 V → 0 V] /negative [0 V → -1 V → 0 V] voltage in M2 mode (Figure S1(b)). While there are negligible changes in the M1 mode under same stimulation (Figure S1(a)), indicating its volatility.

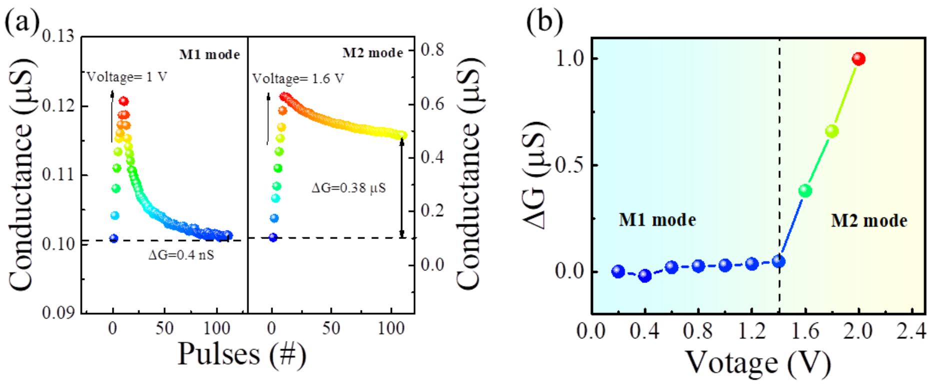


Figure S3. (a) Ten pulses with amplitude of 1 V (left part) and 1.6 V (right part) are applied to the device to record the conductance variation, respectively. The relaxation process was monitored under a read pulse [+0.1 V, 50 ms]. (b) The ΔG as the function of applied voltage.

As shown in Figure 1-3 (a), the conductance variation (ΔG) after the relaxation process depends much on the applied voltage amplitude: a small voltage (e.g. 1 V) result in the volatile switching (ΔG=0.4 nS) in M1, while a large voltage (e.g. 1.6 V) can demonstrate the semi-nonvolatile switching (ΔG=0.38 μS). From Figure S1 (b), the voltage must exceed 1.4 V to convert from M1 mode to M2 mode. In our work, we employed 2 V voltage signal to activate the device to obtain the significantly different memristive behaviors between M1 and M2 mode.

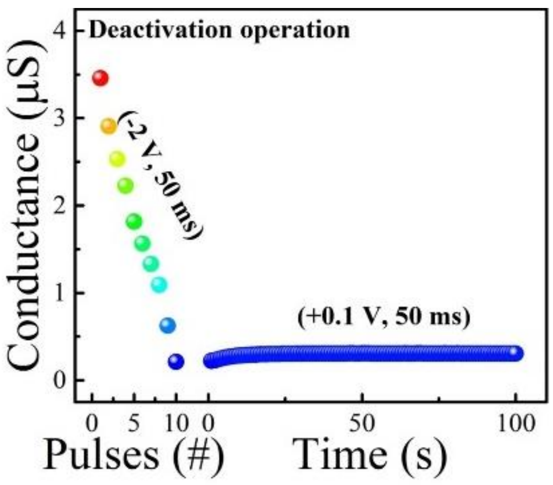


Figure S4. The signal of deactivation operation includes 10 negative pulses [-2 V, 50 ms], which induce the transition from semi-nonvolatile M2 mode to volatile M1 mode. The relaxation process was monitored under a read pulse [+0.1 V, 50 ms].

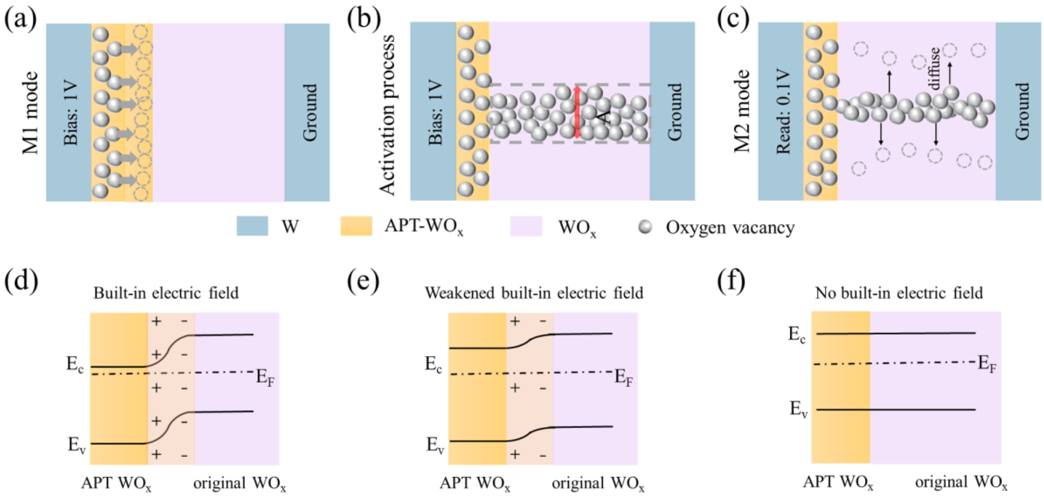


Figure S5. The switching mechanism can be understood according to the schematic diagrams of (a) M1 mode, (b) activation process and (c) M2 mode in the $WO_x$ memristor. (d-f) the corresponding built-in electric field formed at the APT $WO_x$ and $WO_x$ interface in M1 mode, activation process and M2 mode, respectively

For the pristine device in mode M1, a high concentration of $V_O$s was produced on the top interface of W/$WO_x$ by Ar-plasma treatment, leading to high conductance in this region. Thus, a conductance boundary exists between this high-conductance region and the low-conductance region in rest of $WO_x$ film (as shown in Figure S4 (a)) and a built-in electric field at APT $WO_x$ and $WO_x$ interface (as shown in Figure S4(d)). The application of positive pulses [+1V, 50ms] on top electrode can drive the uniform migration of $V_O$s across this boundary, thus increasing the width of high-conductance region and enhancing the device conductance. However, the $V_O$s migration prefers to happen in a limit range near the boundary (B) under such small stimulation, since lower migration barrier at the high Vo concentration and local electric field effect.[1, 2] Those migrated $V_O$s would diffuse back after withdrawing the pulses, leading to the volatile switching in mode M1. Therefore, similar to the models in

literatures,[1,2] the motion of the conductance boundary induced by the migration and diffusion of $V_O$s could explain the switching mechanism of mode M1 in our device.

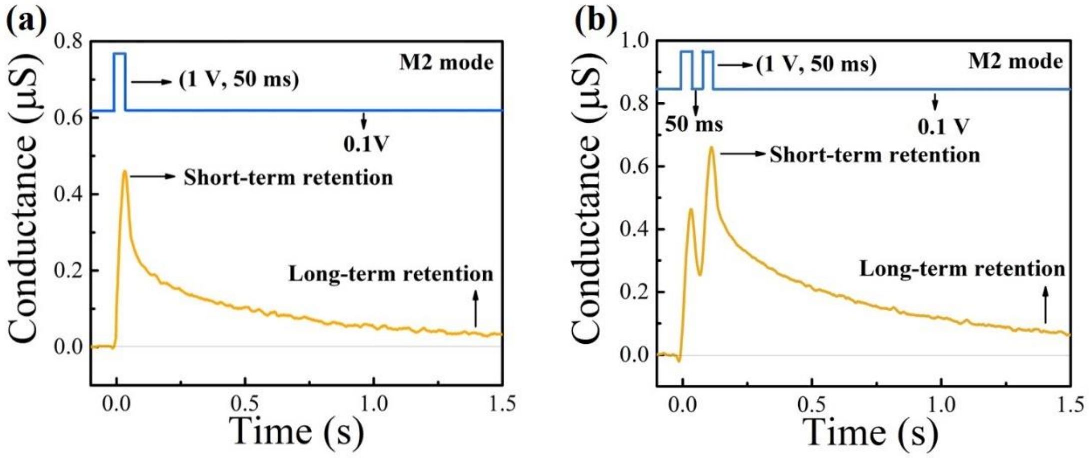


Figure S6. Synaptic functions of (a) EPSC and (b) PPF triggered by a single spike [1 V, 50 ms] and paired spikes in M2 mode. The conductance change can be partially transferred from short-term to long-term retention.

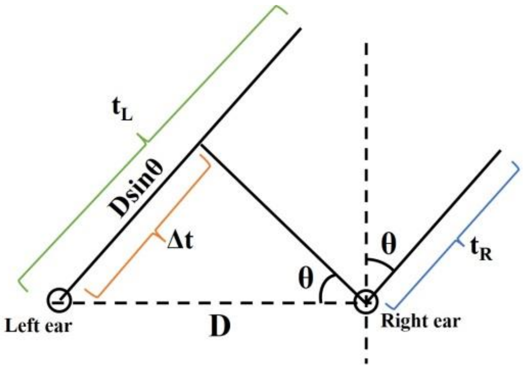


Figure S7. Geometry of the relationship between time difference (*Δt*) and azimuth angle (*θ*).

The time difference (*Δt*), resulting from the sound wave arriving at the near and far ear, can be approximately calculated as follow according to the previous work (as shown in Figure R1-4) [3]:

$$\Delta t = \frac{D\sin\theta}{v}$$

where D is distance between the left and right ear, *θ* is the azimuth angle defined as the angle between the line of sight and the line to the sound source location, v is the speed of sound. We can see that the *Δt* is proportional to the sine of the azimuth angle (Sin*θ*). Herein, when detection angle range from 0º to 90º, *Δt* can be calculated as 0 to 0.45 ms in the external circuit unit (v=340 m/s, D=15 cm). Herein, such electrical signals were converted to match the operating parameter of our memristive device by using a phase difference multiper. The time delay applied to the device (*ΔT*) can be expressed as: *ΔT*=*kΔt* (range from 0~100 ms). $\Delta T$ is proportional to the sin $\theta$.

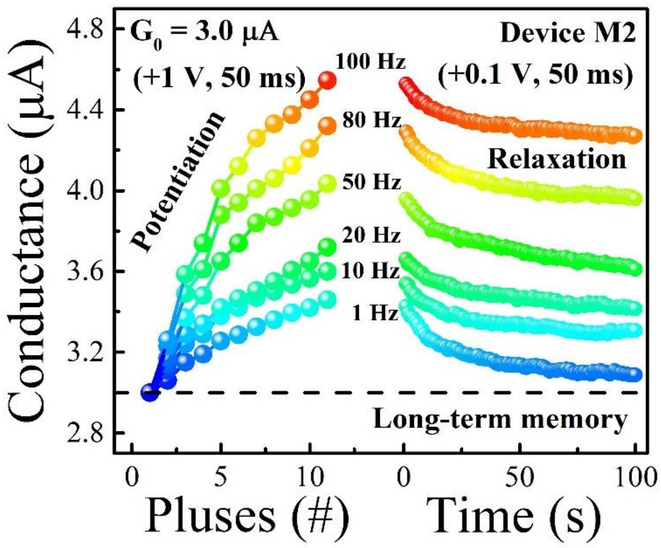


Figure S8. Long-term spike-rate-dependent plasticity (SRDP) is emulated using different frequency spike trains. Spontaneous decay (+0.1 V, 50 ms) of conductance *G* after the potentiation process (+1 V, 50 ms). The basic behavior that higher (lower) spike rate results in larger (smaller) long-term $\Delta G$.

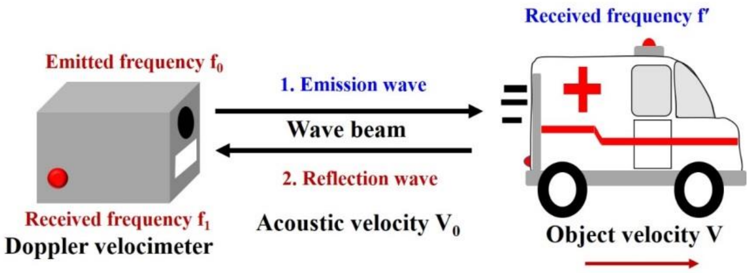


Figure S9. The schematic diagram and measurement process of Doppler velocimeter. The principle of Doppler velocimeter is that when the natural emission frequency wave illuminates the moving object, the reflected or scattered wave will produce Doppler frequency shift due to relative motion.

Take the moving object moving away from the Doppler velocimeter as an example, velocity measurement can be divided into two steps:

1. In the first step, the Doppler velocimeter emits sound waves, and the moving objects receive the sound waves emitted by them. In this process, the Doppler velocimeter is stationary as the wave source, and the moving object moves with the velocity v as the wave receiver. Suppose the emitted frequency of the sound wave from the Doppler velocimeter is $f_0$, the received frequency of the sound wave from the moving object is $f'$, and the propagation speed of the sound wave is $V_0$. The frequency-dependent relationship between the Doppler velocimeter and the moving object is as follows:

$$f' = \frac{V_0 - V}{V_0} f_0 \tag{S1}$$

2. In the second step, the moving object reflects or scatters the sound waves, and the Doppler velocimeter receives the reflected or scattered sound waves. In this process, the moving object acts as a wave source and moves at a velocity V, and the Doppler velocimeter acts as a wave receiver at rest. Let the frequency of the sound wave received by the Doppler velocimeter be

$f_1$, the frequency-dependent relationship between the Doppler velocimeter and the moving object is as follows:

$$f_1 = \frac{V_0}{V_0 + V} f' \qquad \text{(S2)}$$

Substitute equation (S1) into equation (S2) to get:

$$\frac{f_1}{f_0} = \frac{V_0 - V}{V_0 + V} f' \qquad \text{(S3)}$$

Converted from equation (3) to get:

$$V = \frac{f_0 - f_1}{f_0 + f_1} V_0 \qquad \text{(S4)}$$

Because $f_0$ and $V_0$ are known constants, the source velocity detection is only related to the received frequency $f_1$ of Doppler velocimeter.

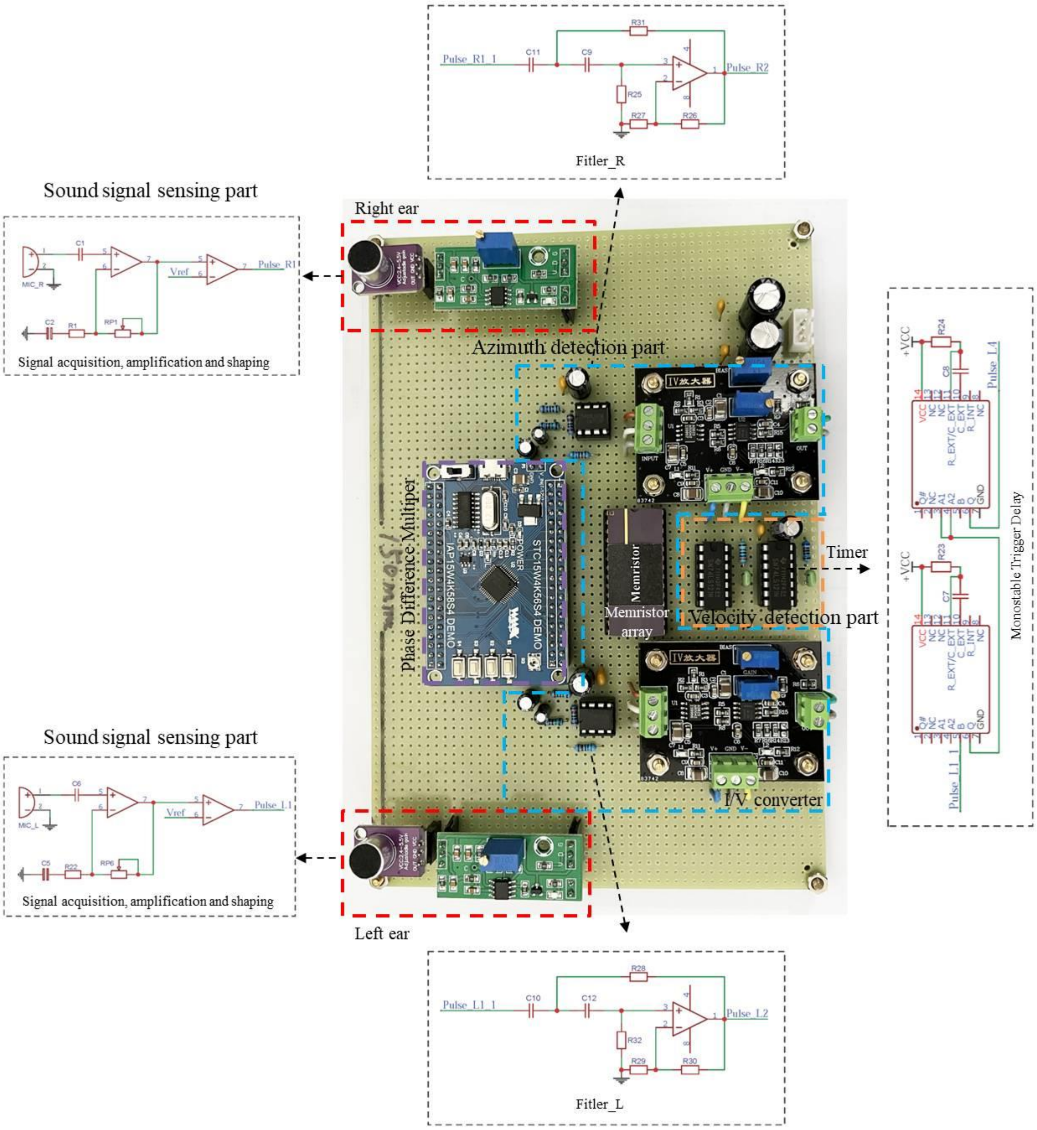


Figure S10. The image of actual circuit to demonstrate the azimuth detection and velocity detection functions. This circuit system includes the sound signal sensing part (the red frame), the azimuth detection part (the blue frame) and velocity detection part (the yellow frame).

Table S1. Comparison of auditory motion perception capabilities implemented on different devices.

| Device structure | Operation Current | Power (/spike) | Azimuth detection | Velocity detection | Ref. |
|---|---|---|---|---|---|
| $Cr/Cu/MoS_2/SiO_2/Si$ | 7.5 pA | 3.8 pW | √ | × | 4 |
| $Ti/HfO_x:Si/TiN$ | 0.1 mA | 100 μW | √ | × | 5 |
| Au/IZGO/Solid-electrolyte | 200 nA | 0.4 μW | √ | × | 6 |
| $TiN/TaO_y/HfO_x/TiN$ | 3 μA | 4.5 μW | √ | × | 7 |
| $TiN/HfO_2/Ti/TiN$ Au/PDVT- | 40 μA | 80 μW | √ | × | 8 |
| 10/Ion-gel/Si | 6 μA | 2.4 μW | √ | × | 9 |
| EMIM:TFSI/Au/P3HT/Au | 0.9 μA | 0.36 μW | √ | × | 10 |
| $Pt/NbO_x/Pt$ | 6 mA | 12 mW | √ | × | 11 |
| $W/APT\text{-}WO_x/WO_x/W$ | 0.15 μA | 120 pW | √ | √ | Our |

**Reference**

[1] Z. Wang, S. Ambrogio, S. Balatti, S. Sills, A. Calderoni, N. Ramaswamy, D. Ielmini, *IEEE Trans. Electron. Devices* **2016**, *63*, 4279.

[2] S. Clima, Y. Y. Chen, R. Degraeve, M. Mees, K. Sankaran, B. Govoreanu, M. Jurczak, S. De Gendt, G. Pourtois, *Appl. Phys. Lett.* **2012**, *100*, 133102.

[3] T.R Letowski and S.T Letowski, "Auditory spatial perception: Auditory localization," Tech. Rep., DTIC Document, May 2012

[4] L. Sun, Y. Zhang, G. Hwang, J. Jiang, D. Kim, Y. A. Eshete, R. Zhao, H. Yang, *Nano Lett.* **2018**, *18*, 3229

[5] W. Wang, G. Pedretti1, V. Milo, R. Carboni, A. Calderoni, N. Ramaswamy, A, S. Spinelli, D. Ielmini, *Sci. Adv.* **2018**, *4*, eaat4752.

[6] Y. He, S. Nie, R. Liu, S. Jiang, Y. Shi, Q. Wan, *Adv. Mater.* **2019**, 31, 1900903.

[7] B. Gao, Y. Zhou, Q. Zhang, S. Zhang, P. Yao, Y. Xi, Q. Liu, M. Zhao, W. Zhang, Z. Liu, X. Li, J. Tang, H. Qian, H. Wu, *Nat. Commun.* **2022**, *13*, 2026.

[8] F. Moro, E. Hardy, B. Fain, T. Dalgaty, P. Clémençon, A. Prà, E. Esmanhotto, N. Castellani, F. Blard, F. Gardien, T. Mesquida, F. Rummens, D. Esseni, J. Casas, G. Indiveri, M. Payvand, E. Vianello, *Nat. Commun.* **2022**, *13*, 3506.

[9] Y. Liu, E. Li, X. Wang, Q. Chen, Y. Zhou, Y. Hu, G. Chen, H. Chen, T. Guo, *Nano Energy* **2020**, *78*, 105403.

[10] G. Liu, Q. Li, W. Shi, Y. Liu, K. Liu, X. Yang, M. Shao, A. Guo, X. Huang, F. Zhang, Z. Zhao, Y. Guo, Y. Liu, *Adv. Funct. Mater.* **2022**, *32*, 2200959.

[11] S. Zhong, Y. Zhang, H. Zheng, F. Yu, .R. Zhao, *Adv. Intell. Syst.* **2022**, *4*, 2200076.
